# Microsatellite Status Detection of Colorectal Cancer: Evaluation of Inconsistency between PCR and IHC

**DOI:** 10.7150/jca.81675

**Published:** 2023-04-17

**Authors:** Jielin Chen, Qijia Yan, Jingyue Sun, Qingyi Wang, Yongguang Tao, Desheng Xiao, Bin Xie

**Affiliations:** 1 Department of Pathology, Xiangya Hospital, Central South University, Changsha, Hunan 410078, China; 2 Department of Pathology, School of Basic Medicine, Central South University, Changsha, Hunan 410078, China; 3 Cancer Research Institute, School of Basic Medicine, Central South University, Changsha, Hunan, 410078, China; 4 Key Laboratory of Carcinogenesis and Cancer Invasion (Central South University), Ministry of Education, Hunan, 410078, China; 5 Key Laboratory of Carcinogenesis (Central South University), Ministry of Health, Hunan, 410078, China

**Keywords:** colorectal cancer, mismatch repair, microsatellite instability, polymerase chain reaction, immunohistochemistry

## Abstract

**Objective:** An essential component of precision medical treatment for colorectal cancer (CRC) is the use of microsatellite state in combination with polymerase chain reaction (PCR) and immunohistochemistry (IHC) as the primary clinical detection methods. Microsatellite instability-high (MSI-H) or mismatch-repair deficiency (dMMR) accounts for about 15% of all CRC patients. Characterized by a high mutation burden, MSI-H is a predictive biomarker of immune checkpoint inhibitors (ICIs). Misdiagnosis of microsatellite status has been shown to be an important cause of resistance to immune checkpoint inhibitors. Therefore, a rapid and accurate assessment of microsatellite status can be beneficial for precision medicine in CRC.

**Methods:** We evaluated the rate of discordance between PCR and IHC detection of microsatellite status from a cohort of patients that had 855 colorectal cancers. PCR-based microsatellite assay was performed using a set of five monomorphic mononucleotide makers (NR-24, BAT-25, CAT-25, BAT-26, MONO-27) and two polymorphic pentanucleotide (Penta D and Penta E). IHC was used to detect the absence of mismatch repair proteins (MLH1, MSH2, MSH6, and PMS2). The inconsistency rates of the two assays were evaluated.

**Results:** Among 855 patients,15.6% (134 to 855) cases were identified as MSI-H by PCR, whereas 16.9% (145 to 855) cases were identified as dMMR by IHC. There were 45 patients with discordant results between IHC and PCR. Of these, 17 patients were classified as MSI-H/pMMR and 28 patients as MSS/dMMR. When the clinicopathological characteristics of these 45 patients were compared to those of the 855 patients, it was found that more patients were younger than 65 years old (80% to 63%), more were male (73% to 62%), more were located in the right colon (49% to 32%), and more were poorly differentiated (20% to 15%).

**Conclusion:** Our study demonstrated a high concordance between the PCR and IHC results. In order to reduce the ineffective treatment of ICIs due to MSI misdiagnosis, the patient's age, gender, tumor location and degree of differentiation should be included in the clinician's selection of MSI testing in colorectal cancer.

## Introduction

In recent years, with the continuous development of medical science, the concept of precision medicine has attracted more and more attention. Precision medicine is based on personal genomic information, combining the patient's living environment and clinical data, while using molecular imaging technology and bioinformatics technology, so as to establish individualized disease prevention and treatment programs and achieve precision diagnosis and treatment 1. In precision medicine, molecular diagnosis leads the way. At present, first-generation sequencing techniques such as real-time fluorescence quantitative polymerase chain reaction (PCR) and immunohistochemistry (IHC) are widely used in the clinic, which brings benefits to many patients.

According to the cancer statistics reported by the American Cancer Society in 2022, colorectal cancer (CRC) ranks third in terms of incidence and mortality, which is a significant component of the cancer burden 2. Microsatellite instability (MSI) is one of the main molecular subtypes of CRC, accounting for 15% of CRC suffers 3-5. Microsatellite is a short tandem repeat sequence consisting of 1-6 nucleotide repeat DNA sequences scattered in the human genome 6. The main system of DNA repair is mismatch repair (MMR) system, which is composed of four DNA mismatch repair proteins, MLH1, MSH2, MSH6, and PMS2, specifically to repair mismatches, deletions, and insertions that occur during cell replication 7, 8. MSI is due to a DNA mismatch repair system deficiency (dMMR). When the MMR system is defective, the repetition length of microsatellites is altered, which leads to the occurrence of a high degree of microsatellite instability 9. Microsatellite instability-high (MSI-H) also minds dMMR tumors have different clinical characteristics and better prognosis than microsatellite stable (MSS) tumors 10-12. Therefore, it is recommended to detect microsatellite instability in all patients with CRC. Recent studies have shown that there are a large number of mononucleotide mutations and frameshift mutations in MSI-H/dMMR tumors, which being characterized by a high tumor mutation load 13. High tumor mutations are considered biomarkers to predict the efficacy of ICIs because of gene mutations that cause tumors to produce new immunogenic antigens. PD1 inhibitors are one of the ICIs. A growing number of researches have shown that patients with MSI-H/dMMR CRC can benefit from PD1 inhibitor therapy 14-17. In 2017, FDA approved PD1 inhibitor pembrolizumab as a first-line agent for the treatment of advanced MSI-H/dMMR CRC 18. As a result, MSI-H/dMMR tumors will become the standard for the use of ICIs in the future. This suggests that the detection of microsatellite instability is an essential part of the precision medicine of CRC.

At present, MSI is mainly diagnosed by polymerase chain reaction (PCR) and immunohistochemistry (IHC). Specific microsatellite repeats (including five monomorphic mononucleotide makers: NR-24, BAT-25, CAT-25, BAT-26, and MONO-27) are amplified by PCR 19-21, and then the size of these microsatellite repeats in tumor and normal tissues were evaluated by capillary electrophoresis. IHC is frequently used as an alternative to PCR to detect the MMR defect by detecting the expression of MMR protein in tumor tissue. The absence of one or more MMR protein expressions is diagnosed as dMMR 22.

MSI-PCR correctly identified 97% of MSI-H when monomorphic mononucleotide makers were used as markers. The correct rate of MMR-IHC was 88.8% 5. Based on the existing studies, the concordance rate between PCR and IHC ranges between 1% and 10% 23-25. Importantly, resistance to immune checkpoint inhibitors in colorectal cancer is associated with misdiagnosis of microsatellite instability 26. Therefore, how to quickly and accurately assess microsatellite status has attracted our attention. In this study, we evaluated the rate of inconsistency between MMR-IHC and MSI-PCR in patients with CRC and analyzed the causes of inconsistency in order to improve the diagnosis of MSI-H/dMMR.

## Materials and Methods

### Patients and tumor characteristics

This large retrospective central study included all 855 patients with CRC who underwent PCR and IHC testing for microsatellite statues at Xiangya Hospital from April 2014 to January 2022. The patient's age, gender, tumor location, and differentiation were included in the retrospective analysis of the data. This study was approved by the Medical Ethics Committee of Xiaoya Hospital, Central South University. Informed consent was waived due to the data already obtained for this retrospective study.

### MSI molecular testing

Genomic DNA was extracted from Formalin-Fixed and Paraffin-Embedded (FFPE) using a DNA isolation kit (AmoyDx 8.0223501X036G). MSI detection kit (AmoyDx 8.0627301X024G) was used to detect five consensus mononucleotide repeats microsatellite markers (NR-24, BAT-25, CAT-25, BAT-26, and MONO-27) in tumor tissue samples and normal tissue samples. At the same time, two pentanucleotide markers (Penta D and Penta E) were detected to determine the same origin of tumor tissue and normal tissue. Then, the PCR products were separated by capillary electrophoresis with ABI3500Dx Genetic Analyzer. The electrophoretic profiles of normal tissues were used as controls: (I) when the size change of two or more monomorphic mononucleotide makers in tumor tissue is greater than or equal to 3bp, it is judged to be MSI-H; (II) When the change in the size of one monomorphic mononucleotide makers fragment in tumor tissue is greater than or equal to 3bp, it is judged to be MSI-L. (III) when the change in the size of no monomorphic mononucleotide makers fragment in tumor tissue is greater than or equal to 3bp, it is judged to be MSS.

### Expression of MMR proteins

Four-micrometer-thick sections were obtained for immunohistochemical studies, which were performed on formalin-fixed, paraffin-embedded tissues using standard peroxidase immunohistochemistry techniques, heat-induced epitope retrieval buffer, and primary antibodies against MMR protein. The antibody includes MLH1 (MBX Biotechnologies ES05), MSH2 (MBX Biotechnologies MX061), MSH6 (MBX Biotechnologies MX056), and PMS2 (MBX Biotechnologies EP51). Lymphocytes in tissue and normal intestinal mucosal epithelial cells were used as a positive control, PBS buffer was used instead of the primary antibody as a negative control, and brown-yellow particles in nuclei were used as positive results. Any nuclear expression in tumor cells is considered to be positive for this MMR protein. One or more MMR proteins with nuclear expression loss in tumor tissues are considered to be dMMR, otherwise, they are considered to be DNA mismatch repair system proficient (pMMR).

### Statistical analysis

IBM SPSS statistics 19 was used for statistical analysis. The coincidence rate of MMR-IHC and MSI-PCR results was calculated. The stratification characteristics of patients were analyzed by the Person chi-square test. When the P value was less than 0.05, the stratification characteristics were considered to be statistically significant.

## Results

### Demographics of CRC patients with different microsatellite status

**Figure 1:**
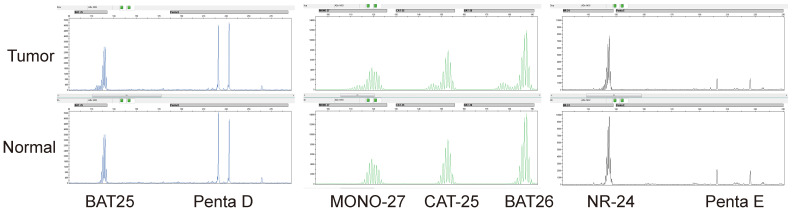
** MSI was detected by multiplex fluorescence PCR combined with capillary electrophoresis.** The normal tissue Penta D and Penta E were both present in the tumor tissue of this patient, indicating that normal and tumor tissues originated from the same patient. The tumor tissues BAT25, MONO-27, CAT-25, BAT26, and NR-24 were all shifted to the left by greater than or equal to 3 bp compared with the normal tissues, indicating that all five monomorphic mononucleotide makers were altered by deletion, and the results were determined to be MSI-H.

**Figure 2:**
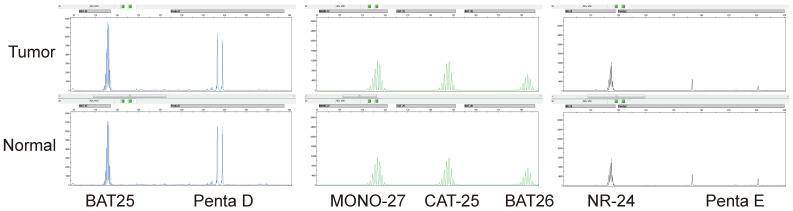
** MSI was detected by multiplex fluorescence PCR combined with capillary electrophoresis.** The normal tissue Penta D and Penta E were present in the tumor tissue of this patient, indicating that normal and tumor tissues originated from the same patient. The tumor tissues BAT25, MONO-27, CAT-25, BAT26, and NR-24 were unchanged compared with normal tissues, indicating that none of the five monomorphic mononucleotide markers were altered by deletion, and the results were judged as MSS.

855 CRCs diagnosed between April 2014 to January 2022 at the XiangYa Hospital were screened for MSI PCR and MMR IHC. According to the statistics, 15.6% (n = 134) patients were diagnosed as MSI-H (Figure 1) and 84.4% (n = 721) patients were identified as MSS (Figure 2) by PCR test, a total of 16.9% (n = 145) patients were identified as dMMR by IHC test, and 83.1% (n = 710) patients were identified as pMMR (Table 1). Since there was no significant difference in clinical features, prognosis, and treatment with immunosuppressant agents between MSI-L as well as MSS patients, they were uniformly classified as MSS in this study. We summarized the clinical and pathological features of these patients (Table 1). Overall, among these 855 patients, the age range spanned 19 to 90 years old, and the median age was 60 years old, with a larger number younger than 65 years, accounting for 63% (n = 542). There were 532 males (59%) and 323 females (41%) (Table 1). The tumors were mainly located in the left colon. 578 (68%) patients had primary tumors in the left colon, while 277 patients (32%) had primary tumors in the right colon (Table 1). The degree of tumor differentiation was moderately differentiated in a total of 663 (77%), which was much higher than 67 (8%) with well differentiation and 125 (15%) with poorly differentiation (Table 1). Compared with the clinical and pathological characteristics of the total of 855 patients, MSI-H/dMMR patients were younger, with a median age of 53 years, with 104/108 patients under 65 years old (78%/74%), while the median age of MSS/pMMR patients was 61 years, and 438/434 MSS/pMMR patients under 65 years old (Table 1). At the same time, right colon cancer accounted for a higher proportion of MSI-H/dMMR patients (67%/59%) and poorly differentiated (48%/30%) (Table 1). The population characteristics of these MSI-H/dMMR patients are consistent with previous studies 27.

### Expression of four mismatch repair proteins

**Figure 3:**
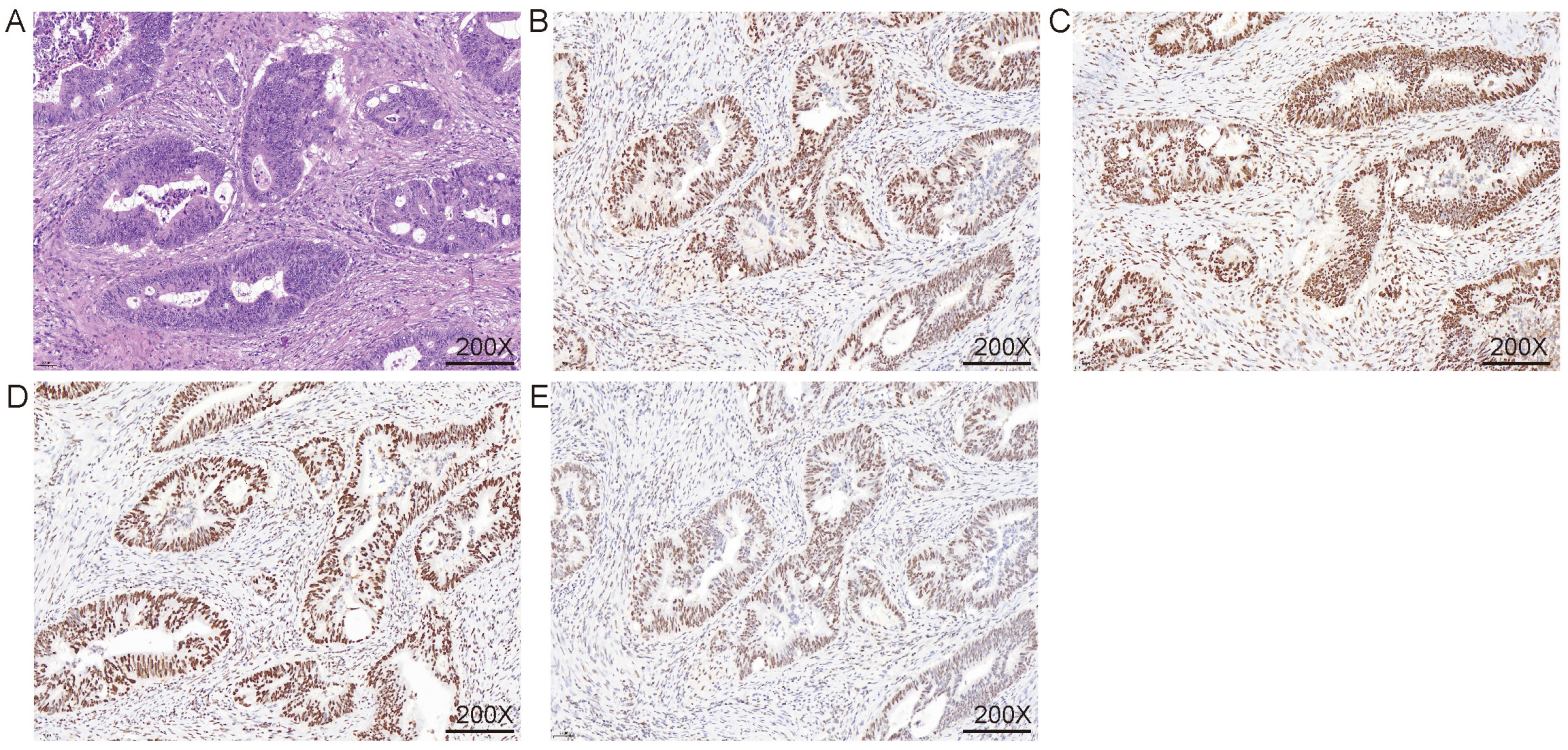
** Immunohistochemical detection of mismatch repair system.** A. HE staining; B. MLH1 IHC; C. MSH2 IHC; D. MSH6 IHC; E. PMS2 IHC. The nuclei of fibroblasts and fibroblasts, lymphocytes, and normal intestinal mucosal epithelial cells in the tissue were colored as positive internal controls.

**Figure 4:**
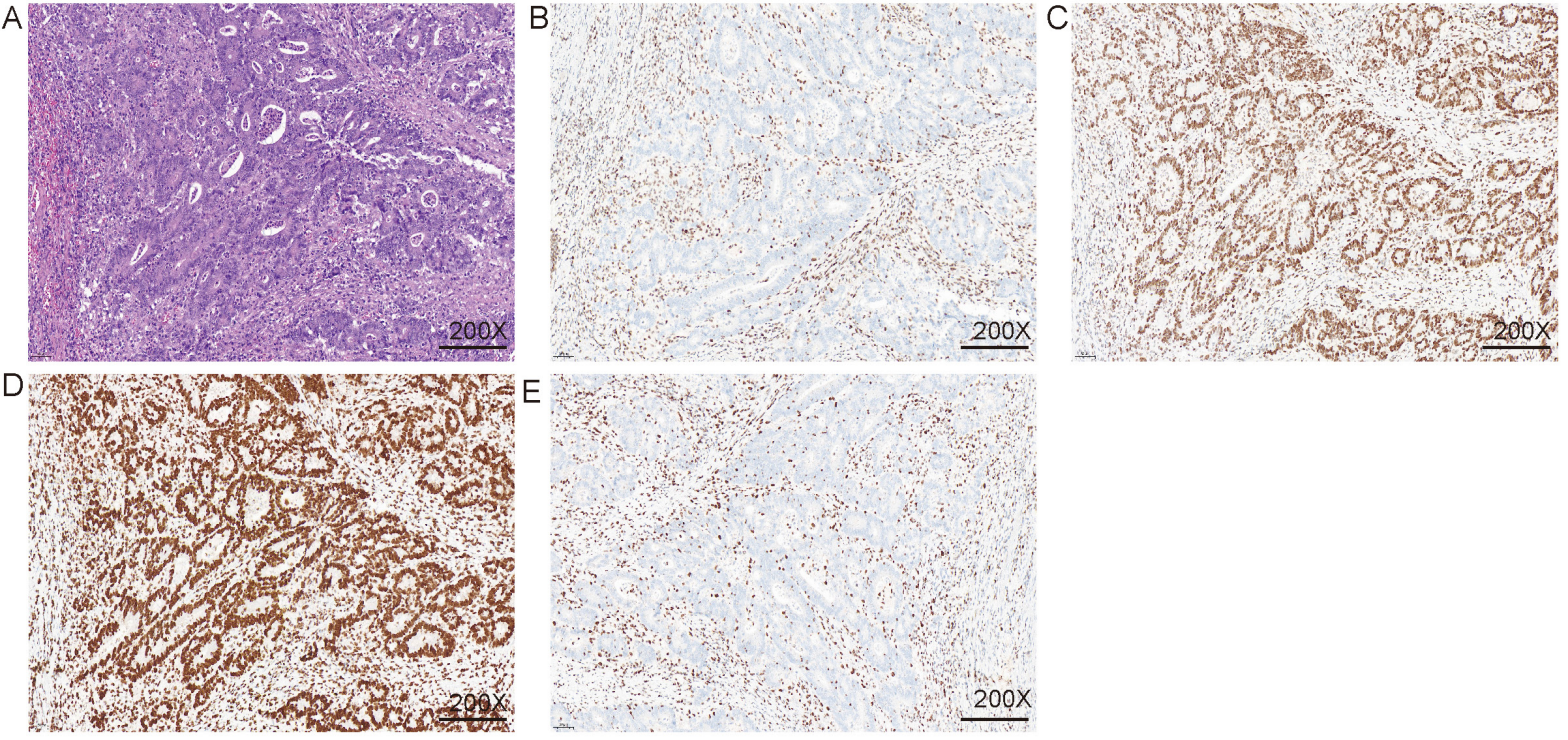
** Immunohistochemical were used to detect MMR.** A. HE staining; B. MLH1 IHC; C. MSH2 IHC; D. MSH6 IHC; E. PMS2 IHC. The nuclei of fibroblasts and fibroblasts, lymphocytes, and normal intestinal mucosal epithelial cells in the tissue were colored as positive internal controls.

The specific expression of all immunohistochemical results, in this case, was counted. Excluding the cases where all four MMR proteins were expressed (Figure 3), the highest proportion was the co-deletion of MSH2 and MSH6, which was 4.95% (n = 42) (Table 2). The second was PMS2 single deletion and common deletion of MLH1 and PMS2 (Figure 4), accounting for 4.6% (n = 39) and 3.9% (n = 33), respectively (Table 2). A total of 19 patients (2.2%) were missing MSH6 alone. Patients with deletion of MLH1 alone and MSH2 alone both accounted for 0.5% of each of the 2 patients (Table 2). All four proteins were missing in two patients (0.2%). Finally, one person each was deficient in MLH1 and MSH2 or MSH6 and PMS2 together (Table 2).

### Comparison of PCR and IHC detection accuracy

PCR is more accurate in MSI detection, it is considered to be the Golden criteria of diagnosis of microsatellite status detection. Therefore, we judge the accuracy of the IHC assay based on the result of PCR. The sensitivity of IHC detection was 87.3%, and the specificity was 96.1% (Table 3). The positive predictive value was 80.7% and the negative predictive value was 97.6% (Table 3). In this study, the correct index of IHC is 0.834 (Table 3).

### Inconsistent rates of PCR and IHC and demographics of inconsistent cases

The statistical results of this study show that the coincidence rate of IHC and PCR is 94.9%. There were 45 patients (5.3%) with inconsistent test results, 17 patients were diagnosed with MSI-H/pMMR and 28 patients were identified with MSS/dMMR (Table 4). Among them, 36 patients were under the age of 65 years old. There were far more males than females, with 73% (n = 33) male patients and 27% (n = 12) female patients (Table 4). 49%/51% (n = 22/23) patients with primary tumors located in the right/left colon. Highly, moderately, and poorly differentiated tumors made up 4% (n = 9), 71% (n = 32), and 20% (n = 9) of the total number of tumors, respectively (Table 4). According to statistical results, these inconsistent patients are younger and are mostly located in the right colon. In addition, among the patients with inconsistent results, there were 11 patients were missing PMS2 expression individually, 5 were deficient in MSH6 alone, 2 were absent in each of MSH2 or MLH1 alone, 5 were co-deleted in MLH1 and PMS2, 2 were co-deleted in MSH2 and MSH6, and one was absent in all four proteins.

## Discussion

The concept of precision medicine, in which individualized treatment plans are tailored to the specificity of the patient, has been widely promoted in the clinic. As one of the main molecular typing of CRC, MSI-H is an important biomarker for clinicians to develop treatment plans. It can not only indicate the prognosis but also predict the efficacy of treatment with 5-FU and ICIs 14-17, 28. A retrospective study confirmed that misdiagnosis of MSI-H/dMMR is one of the causes of drug resistance at ICIs 26. The use of accurate detection methods to evaluate microsatellite status can effectively prevent patients from receiving unnecessary and potentially harmful treatment regimens.

At present, it is generally believed that PCR is the Golden criteria of diagnosis for detecting microsatellite instability in colorectal cancer, and there are several main reasons why it is superior to IHC. First of all, the mistranslation mutation of mismatch repair gene will lead to the loss of protein function without affecting its expression, resulting in PCR detection as MSI-H and IHC detection as pMMR. Second, PCR detection can detect microsatellite instability caused by MSH3 inactivation, whereas conventional IHC detection does not include MSH3 as a protein detection index, resulting in missed diagnosis 29. Finally, radiotherapy or chemotherapy will reduce or lose the expression of MLH1 or MSH6, which will affect the results of IHC but not the results of PCR 30, 31. Therefore, this study is based on PCR to evaluate the results of IHC.

Comparing the results of the two assays, the inconsistency rate was 5.3%. When tested only with IHC, 17 patients identified with MSI-H were classified as pMMR. When using PCR alone, 28 dMMR patients were classified as MSS. All of these 45 patients may have made incorrect decisions about immunosuppressive therapy due to the limitations of the test, thus delaying the timing of treatment. Combined with the results of others, we analyzed the reasons for the inconsistent results of this study and the hints of the clinicopathological characteristics of these inconsistent patients on the choice of detection methods.

In our study, FFPE was used for MSI/MMR testing, excluding inadequate sampling of tissue specimens such as rapidly frozen tissue specimens and cytological specimens 32. It is reported that the expression of MLH1 and MSH6 is missing after cisplatin treatment 30, 31. Therefore, the patients included in this study did not neoadjuvant radiotherapy before detecting microsatellite status. In addition, the physicians who analyzed the results of PCR or IHC testing in this study were not informed of the results of the microsatellite status of another test diagnosis, thus avoiding bias in the interpretation of the results. We ensured that the immunohistochemical staining results were interpreted by two experienced pathologists, respectively, and disputed results were co-diagnosed by a third pathologist to ensure the accuracy of the IHC test.

Assessment of microsatellite status by PCR relies heavily on microsatellite markers. Currently, although the selection of the most suitable microsatellite markers for MSI is still controversial, the use of monomorphic mononucleotide makers to detect microsatellite status to evaluate microsatellite status has gained consensus. In 2004, Promega developed a panel that is more sensitive and specific, using five monomorphic mononucleotide makers (BAT-25, BAT-26, NR-21, NR-24, and MONO-27) and two pentanucleotide markers (Penta C and Penta D) as homology controls 19. After that, studies have found that CAT-25 is a sensitive and specific marker for the detection of MSI, and there was 100% concordance between the use of CAT-25 and the use of the Bethesda panel for the detection of MSI-H colorectal cancer 21. Therefore, we combine both, using five monomorphic mononucleotide makers (NR-24, BAT-25, CAT-25, BAT-26, and MONO-27) and simultaneously testing two pentanucleotide markers (Penta D and Penta E) to improve the accuracy, specificity, and reliability of the assay. Among the patients with inconsistent tests, 17 MSI-H patients had no loss of MMR protein expression. This was due to the ability to synthesize nonfunctional mismatch repair proteins despite having mutated at the DNA level of the MMR gene, thus failing to detect defects in the mismatch repair system by IHC 27.

Of the other 28 patients tested with dMMR/MSS, 11 had a single deletion of PMS2, and 5 had a deletion of MSH6 alone. In the mismatch repair system, mutations in the MMR gene interfere with protein dimerization, resulting in loss of protein expression after heterodimer proteolysis. MLH1 and PMS2 form heterodimers, while MSH2 can form heterodimers with MSH6. Because MSH3 and PMS1 can substitute for PMS2 and MSH3 can substitute for MSH6, MLH1, and MSH2 can often remain stable in the absence of dimer partners 7, 8, 28, 33, 34. Therefore, the loss of MLH1 or MSH2 is often accompanied by the loss of PMS2 and MSH6. In contrast, when PMS2 and MSH6 were mutated, only the affected protein expression was lost. In the case of PMS2 and MSH6 deletion alone, because the role of chaperone proteins is compensated by other proteins, the heterodimer complex can still play the function of mismatch repair, thus maintaining microsatellite stability and being MSS by PCR detection. One study classifies patients without PMS2 or MSH6 as a special type of dMMR (regardless of microsatellite status), which shows that these patients have a high level of genetic susceptibility and can benefit from treatment with ICIs 35. The number of cases in which this conclusion was reached is small, however, and more studies are needed to confirm this conclusion. For these 16 patients, the decision to treat with ICIs should be made in conjunction with other test results and the clinician's experience, rather than excluding them from treatment with ICIs based on PCR results alone.

We attempted to correlate the misdiagnosis of microsatellite status by analyzing whether there were clinical or pathological features associated with it. Patients with inconsistent results are often younger and more likely to be located in the right colon (Table 4). Although there are more men and poorly differentiated in terms of the number of cases, there is no statistically significant difference. Combining the results of the current study with previous studies, it is not difficult to conclude that MSI-H patients are younger, more male, and are common in right colon cancer and poorly differentiated cancer. To avoid misdiagnosis, it is recommended that when patients have the above clinicopathological features, particularly the age and location characteristics, a combination of PCR and IHC testing protocols be used to avoid misdiagnosis and thus influence the clinician's judgment of the treatment plan.

Some studies have shown that BRAF mutation is closely related to microsatellite status. Sporadic CRC is usually caused by hypermethylation of MLH1 promoter, resulting in loss of both MLH1 and PMS2 expression 36, 37. Hypermethylation of MLH1 promoter region is associated with BRAF 38. However, although different studies have tried to determine the prognostic value of BRAF V600E mutation together with microsatellite status, none of them have reached a definitive conclusion 39-41. In this study, MLH1 methylation and BRAF V600E mutation were not included in the study for economic consideration.

In recent years, tumor second-generation sequencing technology has begun to be used to examine tumor microsatellite status, showing higher sensitivity and specificity, while being more useful in predicting the efficacy of immune checkpoint inhibitors to determine individualized treatment regimens 42-44. However, due to the economic burden and technical complexity, this technology needs further experimentation and improvement before it enters the clinic. This study pays more attention to comparing the existing detection methods, to provide a reference for the selection and diagnosis of clinicians.

In conclusion, our research showed that the inconsistent rate of using PCR and IHC to detect microsatellite status is low, IHC can be used as a tool for screening, but the preferential use of PCR assay is still recommended in the case of technical support. When the patient has the following clinical and pathological features: 1. Age < 65 years old; 2. Male; 3. Right colon cancer; 4. For poorly differentiated cancer, it is recommended to use both IHC and PCR to determine the microsatellite status. Since MSI-H/dMMR is suggestive of a favorable outcome with immune checkpoint inhibitors. Clinicians should be aware of the limitations of these two detection methods so as to avoid errors in individualized treatment schemes caused by misdiagnosis.

## Figures and Tables

**Table 1:** The clinical characteristics of MMR states were detected by PCR and IHC

Characteristic	Total (n=855) n (%)	PCR		P.value	IHC		P.value
		MSI-H (n=134) n (%)	MSS (n=721) n (%)		dMMR (n=145) n (%)	pMMR (n=710) n (%)	
**Age(years)**							
**Median(range)**	60(19-90)	53(19-82)	61(26-90)		53(19-82)	61(26-90)	
<65	542(63)	104(78)	438(61)	P<0.01	108(74)	434(61)	P=0.002
≥65	313(37)	30(22)	283(39)		37(26)	276(39)	
**Sex**							
Male	532(62)	79(59)	453(63)	P=0.396	86(59)	446(63)	P=0.427
Female	323(38)	55(41)	268(37)		59(41)	264(37)	
**Primary site**							
Rt. colon	277(32)	90(67)	187(26)	P<0.01	86(59)	191(27)	P<0.01
Lt. colon	578(68)	44(33)	534(74)		59(41)	519(73)	
**Differentiation**							
Well Differentiated	67(8)	13(10)	54(7)	P<0.01	15(10)	52(7)	P<0.01
Moderately Differentiated	663(77)	73(54)	590(82)		87(60)	576(81)	
Poorly Differentiated	125(15)	48(36)	77(11)		43(30)	82(12)	

MMR, mismatch repair; PCR, polymerase chain reaction; IHC, immunohistochemistry; MSI, microsatellite instability; MSS, microsatellite stable; dMMR, DNA mismatch repair system deficient; pMMR, DNA mismatch repair system proficient.

**Table 2:** The specific expression of MMR was detected by IHC.

Maker	MLH1	MSH2	MSH6	PMS2	n (%)
**Expression**	(-)	(+)	(+)	(-)	33(3.9)
	(+)	(-)	(-)	(+)	42(4.9)
	(+)	(+)	(-)	(-)	1(0.1)
	(-)	(-)	(+)	(+)	1(0.1)
	(-)	(+)	(+)	(+)	4(0.5)
	(+)	(-)	(+)	(+)	4(0.5)
	(+)	(+)	(-)	(+)	19(2.2)
	(+)	(+)	(+)	(-)	39(4.6)
	(-)	(-)	(-)	(-)	2(0.2)
	(+)	(+)	(+)	(+)	710(83)

MMR, mismatch repair; IHC, immunohistochemistry.

**Table 3:** Difference and consistency of microsatellite instability detected by IHC and PCR.

IHC	PCR	
	MSI-H (n=134)	MSS (n=721)
dMMR (n=145)	117	28
pMMR (n=710)	17	693
Sensitivity of IHC	87.3% (117/134)	
Specificity of IHC	96.1% (693/721)	
Positive predictive value of IHC against PCR	80.7% (117/145)	
Negative predictive value of IHC against PCR	97.6% (693/710)	
Concordance between IHC and PCR	94.7% (810/855)	
Youden Index of IHC	0.834	

PCR, polymerase chain reaction; IHC, immunohistochemistry; MSI, microsatellite instability; MSS, microsatellite stable; dMMR, DNA mismatch repair system deficient; pMMR, DNA mismatch repair system proficient.

**Table 4:** The clinical characteristics of Inconsistent typing.

Characteristic	Total (n=855) n (%)	Typing		P.value
		Consistent typing (n=45) n (%)	Inconsistent typing (n=810) n (%)	
**Age(years)**				
**Median(range)**	60(19-90)	53(27-76)	60(19-90)	
<65	542(63)	36(80)	506(62)	P=0.017
≥65	313(37)	9(20)	304(38)	
**Sex**				
Male	532(62)	33(73)	499(62)	P<0.01
Female	323(38)	12(27)	311(38)	
**Primary site**				
Rt. colon	277(32)	22(49)	255(31)	P=0.015
Lt. colon	578(68)	23(51)	555(69)	
**Differentiation**				
Well Differentiated	67(8)	4(9)	63(8)	P<0.01
Moderately Differentiated	663(77)	32(71)	631(78)	
Poorly Differentiated	125(15)	9(20)	116(14)	

MMR, mismatch repair; PCR, polymerase chain reaction; IHC, immunohistochemistry; MSI, microsatellite instability; MSS, microsatellite stable; dMMR, DNA mismatch repair system deficient; pMMR, DNA mismatch repair system proficient.
